# Adipokines demonstrate the interacting influence of central obesity with other cardiometabolic risk factors of metabolic syndrome in Hong Kong Chinese adults

**DOI:** 10.1371/journal.pone.0201585

**Published:** 2018-08-16

**Authors:** Rashmi Supriya, Bjorn T. Tam, Angus P. Yu, Paul H. Lee, Christopher W. Lai, Kenneth K. Cheng, Sonata Y. Yau, Lawrence W. Chan, Benjamin Y. Yung, Sinead Sheridan, Parco M. Siu

**Affiliations:** 1 Department of Health Technology and Informatics, Faculty of Health and Social Sciences, The Hong Kong Polytechnic University, Hung Hom, Kowloon, Hong Kong; 2 School of Public Health, Li Ka Shing Faculty of Medicine, The University of Hong Kong, Pokfulam, Hong Kong; 3 School of Nursing, Faculty of Health and Social Sciences, The Hong Kong Polytechnic University, Hung Hom, Kowloon, Hong Kong; 4 Department of Rehabilitation Sciences, Faculty of Health and Social Sciences, The Hong Kong Polytechnic University, Hung Hom, Kowloon, Hong Kong; East Tennessee State University, UNITED STATES

## Abstract

**Objective:**

Metabolic syndrome (MetS) or prediabetes is a complex disorder that is defined by a clustering of cardiometabolic risk factors, including obesity, hypertriglyceridemia, reduced high-density lipoprotein (HDL) cholesterol, hypertension, and insulin resistance. Among cardiometabolic risk factors, central obesity plays a key role in the development of MetS through alterations in the secretion of adipokines and interacts with other MetS risk factors to unfavorably influence overall cardiometabolic risk. Obesity has grasped epidemic proportions in Asia, which has the highest number of people with diabetes in the world. But, the importance of central obesity in the clustering of all four MetS risk factors or vice versa in predicting severity of MetS has not yet been investigated in Asian population. Therefore, the present study examined the influence of central obesity on circulating levels of adipokines through its interaction with the clustering of cardiometabolic risk factors of MetS including hyperglycemia, hypertriglyceridemia, dyslipidemia and hypertension in Hong Kong Chinese adults.

**Subjects:**

Blood samples from 83 Hong Kong Chinese adults, who were previously screened for MetS according to the guideline of the United States National Cholesterol Education Program Expert Panel Adult Treatment Panel III criteria were selected. Insulin and adipokines, including visfatin, chemerin, plasminogen activator inhibitor-1 (PAI-1), resistin, C-C motif chemokine ligand 2 (CCL-2), interleukin-6 (IL-6), interleukin-8 (IL-8), interleukin-10 (IL-10), tumour necrosis factor-α (TNF-α), leptin and adiponectin were assessed.

**Results:**

The interacting effect of central obesity with all of the other four MetS risk factors increased the proinflammatory status of adipokines (TNF-α, leptin) and decreased the anti-inflammatory status of adipokine (adiponectin).

**Conclusion:**

Our results indicate that the inflammatory status of MetS may be more severe in the presence of central obesity. Adipokines, as biomarkers for pathophysiological changes, may help to improve early patient identification and to predict MetS-associated morbidity and mortality.

## Introduction

Metabolic syndrome (MetS) is a complex disorder that is defined by the clustering of cardiometabolic risk factors, including obesity, hypertriglyceridemia, reduced high-density lipoprotein (HDL) cholesterol, hypertension, and insulin resistance that together, culminate in an increased risk of type 2 diabetes mellitus (T2D) and cardiovascular disease (CVD) [1]. Obesity, and in particular central obesity, plays an important role in the development of MetS [2] and is associated with an increased risk of cardiovascular disorders, T2D and certain cancers [3]. The prognostic importance of central obesity has been recognized by the International Diabetes Federation which includes central obesity as the *sine qua non* for diagnosing MetS [4]. Central obesity has been associated with an increased risk of CVD in subjects with or without MetS [5]. Intra-abdominal adiposity interacts with other MetS risk factors to unfavorably influence overall cardiometabolic risk. The Quebec Health Survey analyzed the relationship between indices of obesity, blood pressure (BP) and hyperinsulinemia and found that variations in waist circumference explained the associations among obesity, hypertension and insulin resistance [6]. Moreover, intra-abdominal adiposity has been allied with adverse changes in lipid profile in older people before and after adjustment for overall adiposity [7].

Obesity has grasped epidemic proportions in Asia. Asian countries are faced with serious burdens of obesity-related disorders such as diabetes, hypertension, and cardiovascular diseases, which develop at a younger age than in Western populations [8]. For instance, review data in epidemiologic trends revealed that people of Asian ancestry are at higher risk of developing T2D and are more likely to develop obesity-related diseases even at a lower body mass index (BMI) as compared to Europeans [6]. At present, >60% of the world’s diabetic population resides in Asian countries [9]. The higher risk of diabetes in Asians (especially South Asians) may be partly attributed to the fact that they are more central obese but have less muscle, which together cause insulin resistance [10]. Relatively few cross-sectional studies conducted in Hong Kong and Taiwanese populations have shown that central obesity is an independent determinant of insulin resistance and other metabolic risk factors. On the other hand, numerous studies have shown that, while central obesity is important, it is not an essential component of predicting T2D or CVD incidence, especially in Asian populations [11,12]. For instance, the incidence of T2D with or without central obesity was similar in Chinese hypertensive individuals (n = 595) with the same number of MetS risk factors [11]. Another study conducted in Japanese Americans (n = 116) reported that the presence of MetS independently predicted the development of T2D, irrespective of the presence of central obesity [12]. Moreover, non-obese people with ≥ 3 MetS risk factors have been shown to have an equal or slightly higher risk of cardiovascular mortality and renal dysfunction than obese people with ≥ 3 MetS risk factors [13,14]. Nonetheless, these findings are difficult to interpret as the two or more MetS risk factors other than central obesity in the selection of their subjects with MetS was not specified. Interestingly, interventions directed solely to only one of the MetS risk factors in non-obese people at risk of CVD have been proposed to have relatively less beneficial effects on CVD risk reduction. For example, interventions directed solely to lowering blood pressure have been shown to have relatively little beneficial effect reducing risk of ischemic heart diseases due to the presence of hyperinsulinemia with hypertension [15]. In contrast, in the Copenhagen Male Study (n = 5249, mean age = 48 years), it was reported that the dyslipidemia was an important risk factor of ischemic heart disease in individuals with MetS [16]. The same research group also found that BP level was less predictive of the risk of ischemic heart disease in individuals (n = 2906 men, mean age 63 years) with dyslipidemia in MetS compared to those without dyslipidemia [17]. While the aforementioned studies indicate that in non-obese individuals with MetS targeting all of the four MetS risk factors may be important to prevent CVD or T2D risk, the importance of central obesity in the clustering of all four MetS risk factors or vice versa in predicting severity of MetS has not yet been investigated.

Obesity occurs in association with the enlargement of white adipose tissue and is often emphasised in the pathogenesis of MetS [4,18,19]. Specifically, excess intra-abdominal obesity has the potential to influence cardiometabolic risk directly, through alternations in the secretion of adipokines [20]. Adipose tissue is primarily composed of a stromal vascular fraction and adipocytes. The stromal vascular fraction consists of preadipocytes, fibroblasts, endothelial cells and immune cells, including B cells, T cells, and macrophages [21] that play critical roles in various stages of obesity. In a healthy state, adipocytes and M2 macrophages secrete adiponectin and interleukin-10 (IL-10), which assist in anti-inflammatory regulation and tissue repair [22,23]. On the other hand, during the preliminary stage of obesity, the positive energy balance increases the size of adipocytes. These enlarged adipocytes secrete pro-inflammatory adipokines, such as leptin [24], interleukin-6 (IL-6) [25], tumour necrosis factor-α (TNF-α) [26], chemokine (C-C motif) ligand 2 (CCL2) [27], and chemerin, which enhance M1 macrophage chemotaxis. In advanced stages of obesity, the obese adipose tissue activates CD8^+^ T cells, which recruit monocytes and stimulate the pro-inflammatory M1 macrophages in the adipose tissue respectively. Subsequently, large numbers of migrated M1 macrophages suppress the anti-inflammatory effects of M2 macrophages, promote systemic inflammation, contributing to MetS [28,29].

Fluctuations in circulating adipokines linked to MetS have been reported in Asian populations [30–32]. Researchers suggested that the aggregation of MetS risk factors decreases serum adiponectin level and increases leptin and female RBP-4 levels. Intriguingly, adipokines were considerably related to MetS component aggregation [33]. Studies suggested that adipokines may be the main factors involved in the pathogenesis of MetS in Asian populations and may serve as early therapeutic targets and prognostic markers for the development of MetS [33]. A study reported that individuals with elevated levels of more than two pro-inflammatory adipokines had a higher MetS prevalence compared to those individuals with elevated levels of less than two pro-inflammatory adipokines [30]. Additionally, individuals with reduced levels of anti-inflammatory adipokines had a higher prevalence of MetS compared to those with elevated levels of anti-inflammatory adipokines [30]. In another study, the prevalence of MetS was 1.49 times higher in individuals with low adiponectin, an anti-inflammatory adipokine, and high retinol binding protein 4, a pro-inflammatory adipokine, compared to individuals with low retinol binding protein 4 and high adiponectin [34]. Thus, a single adipokine may not induce MetS but the interaction between pro-inflammatory and anti-inflammatory adipokines contributes to systemic metabolic abnormalities while more importantly, increases in perivascular and visceral obesity disrupts the equilibrium between pro-inflammatory and anti-inflammatory adipokines.

The importance of controlling adipokines-induced inflammation in MetS patients to prevent CVD risk has been suggested in Asian and non-Asian populations [35]. Central obesity has a potentially unifying pathogenetic role that is strongly associated with inflammation and oxidative stress leading to MetS [36]. Biomarkers for these clinical and pathophysiological changes have strong potential to improve early patient identification and predict MetS-associated morbidity and mortality. We hypothesised that central obesity plays a unique role in mediating circulatory levels of adipokines through its interaction with the clustering of the other four MetS cardiometabolic risk factors, including hypertriglyceridemia, reduced HDL cholesterol, hypertension, and high fasting glucose. The present study was specifically designed to examine the importance of central obesity on circulating levels of adipokines in the clustering of all of the other four metabolic risk factors in an Asian population.

## Materials and methods

**Fig 1:**
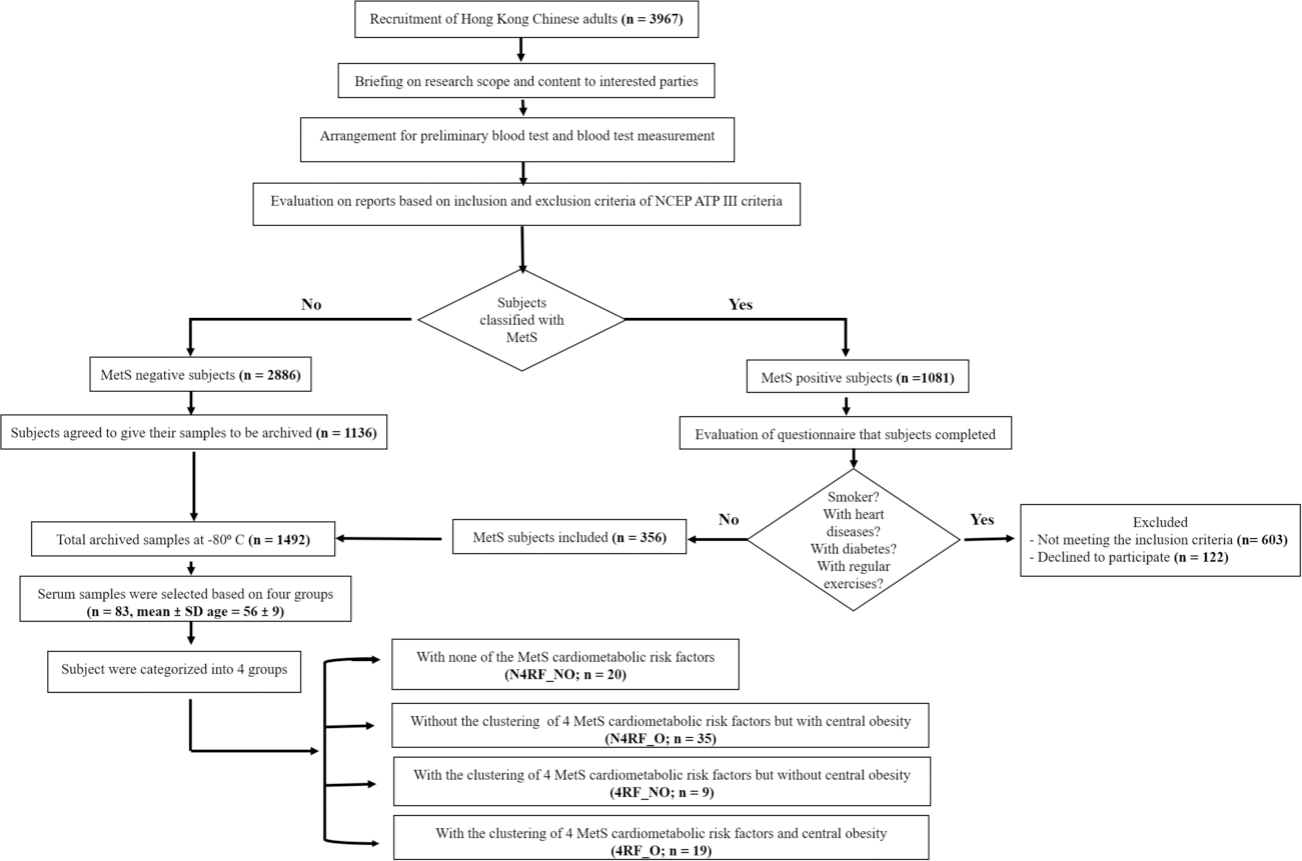
Flowchart of the study design.

MetS risk factors data and serum samples of Hong Kong Chinese adults of both sexes (N = 83, mean±SD age = 56±9.1) were retrieved from a total of 1,492 archived blood samples of participants screened for MetS parameters defined by NCEP-ATP III [22]. In this study, inclusion of subjects was based on 4 groups: 1) subjects with none of the NCEP-ATP III-defined MetS cardiometabolic risk factors, 2) subjects with only central obesity, 3) subjects without central obesity and with the other 4 MetS cardiometabolic risk factors, and 4) subjects with all five cardiometabolic risk factors of MetS. We adopted a 2 x 2 factorial design with central obesity and/or 4 other MetS cardiometabolic risk factors (high systolic and diastolic BP, elevated fasting blood glucose, high triglycerides, and low HDL). All subjects were screened for MetS according to the diagnostic guideline of the NCEP-ATP III criteria [37]. People diagnosed with MetS had more than two of following characteristics: (1) central obesity (waist circumference exceeds 90 or 80 cm for Asian male and female, respectively), (2) hypertension (systolic pressure equals or exceed 130 mmHg, or diastolic pressure equals or exceeds 85 mmHg), (3) elevated blood glucose (fasting glucose level equals or exceeds 5.5 mmol/L [100 mg/dL]), (4) elevated plasma triglycerides (level equals or exceeds 1.70 mmol/L [150 mg/dL]), and (5) low level of HDL-C (level equals or is less than 1.0 mmol/L [40 mg/dL] for male and 1.3 mmol/L [50 mg/dL] for female) are regarded as MetS positive. Participants with severe or acute CVD, post-stroke, neuromusculoskeletal illness, dementia or mental disorders, acute medical illness, symptomatic heart or lung diseases, osteoarthritis or pulmonary illness, severe rheumatoid arthritis, smoker and participants who were immobile, or under treatment for metabolic abnormalities were excluded from the study. Subject participation was voluntary and written informed consent was obtained before this study. Human research ethics approval was provided by the Human Subjects Ethics Subcommittee of the Hong Kong Polytechnic University (HSEARS20150205001). A flowchart explaining the study procedure is presented in Fig 1.

### Selection of subjects and group assignment

In this study, “4RF” was used to identify the subjects with the clustering of all 4 MetS cardiometabolic risk factors (i.e., elevated BP, increased fasting glucose, increased blood triglycerides, and reduced HDL). Subjects without the clustering of all 4 MetS cardiometabolic risk factors were identified as "N4RF”. In addition, central obesity was specifically investigated as an independent factor. Subjects with central obesity were identified as "O", whereas subjects without central obesity were indicated as "NO”. All subjects were divided into 4 groups including subjects with none of the MetS cardiometabolic risk factors (N4RF_NO; n = 20), subjects without the clustering of 4 MetS cardiometabolic risk factors but with central obesity (N4RF_O; n = 35), subjects with the clustering of 4 MetS cardiometabolic risk factors but without central obesity (4RF_NO; n = 9), and subjects with the clustering of 4 MetS cardiometabolic risk factors and central obesity (4RF_O; n = 19).

### Measurements of cardiometabolic risk factors of MetS

All MetS diagnostic parameters were measured by trained research personnel. For central obesity, waist circumference was measured using inelastic measuring tape on the bare skin region between the lowest rib and superior border of the iliac crest. Systolic and diastolic BP was measured using an electronic BP monitor (Accutorr Plus, Datascope) over the brachial artery region on the right arm after 5 minutes of rest, with the arm supported at the level of the heart using an appropriately sized cuff. Fasting venous blood samples were collected after at least 10 hours of fasting by certified phlebotomists. Fasting blood glucose, blood triglycerides, and blood HDL-cholesterol were determined using an automatic clinical chemistry analyser (Architect CI8200, Abbott Diagnostics) in an accredited medical laboratory. A detailed explanation of the methods used for the assessment of MetS cardiometabolic risk factors has been previously described [18].

### Measurements of adipokines and insulin

Commercially available enzyme-linked immunosorbent assay (ELISA) kits were used to perform the biochemical measurements of adipokines and insulin according to the manufacturer’s instructions (visfatin kit was from BioVision; chemerin, PAI-1, resistin, CCL-2, IL-6, IL-8, IL-10 and TNF-α kits were from R&D; leptin, adiponectin and insulin kits were from Thermo Fisher Scientific). The coefficient of variability (CV) for the ELISA kits were shown as follows: visfatin (intra-assay: 4.4–8%; inter-assay: 8.2%), chemerin (intra-assay: 3.9%; inter-assay: 7.3%), PAI-1 (intra-assay: 6.8%; inter-assay: 7%), resistin (intra-assay: 4.7%; inter-assay: 8.4%), CCL-2 (intra-assay: 5%; inter-assay: 5.1%), IL-6 (intra-assay: 2.6%; inter-assay: 4.5%), IL-8 (intra-assay: 4.7–6.7%; inter-assay: 5.8–7.7%), IL-10 (intra-assay: 3.7%; inter-assay: 6.9%), TNF-α (intra-assay: 4.9–7.8%; inter-assay: 4.7%-5.8%), leptin (intra-assay: 3.9%; inter-assay: 5.3%), adiponectin (intra-assay: 3.8%; inter-assay: 5.5%), and insulin (intra-assay: 4.8%-6%; inter-assay: 8.1% to 9%). All measurements were performed in duplicates or triplicates. Seven controls (one blank, two at lower concentrations, two at medium concentrations, and two at higher concentrations) were also quantified in duplicates to check for the reproducibility of measurements and confirm acceptable reproducibility.

### Statistical analysis

Data are expressed as the mean ± standard deviation. The Shapiro-Wilk test was adopted to check the distribution of the data. The data of all adipokines and insulin followed non-normal distribution. Therefore, generalised estimating equation was adopted to analyse the data, where the adipokines and insulin were considered as dependent variables. Central obesity and the clustering of 4 MetS risk factors were considered as the two independent factors for calculating the interaction effect. Interaction effect between central obesity and the clustering of 4 MetS risk factors on adipokines was also analysed by adjusting age and sex as covariates. The Kruskal-Wallis test followed by post hoc tests with Dunn-Bonferroni correction were used to analyse multiple group-wise comparisons and baseline comparisons. Spearman’s correlation analysis was performed to examine the correlations between the peptide concentrations and the MetS parameters. Statistical significance was accepted at P < 0.05 with confidence intervals of 95%. All statistical analyses were performed using the Statistical Package for the Social Sciences version 22 for Windows.

## Results

### Comparison of participant baseline characteristics

No significant differences were observed in sex and age among all the four groups. Baseline comparisons of all the five MetS risk factors between groups were also made. The summary of the participant characteristics is presented in Table 1.

**Table 1:** Baseline characteristics of sex, age and cardiometabolic risk factors in the following 4 groups: 1) subjects with none of the cardiometabolic risk factors (N4RF_NO; n = 20), 2) subjects with only central obesity without the other 4 MetS cardiometabolic risk factors (N4RF_O; n = 35), 3) subjects without central obesity but with the other 4 MetS cardiometabolic risk factors (4RF_NO; n = 9), and 4) subjects with all five MetS cardiometabolic risk factors (4RF_O; n = 19). Data are expressed as mean ± standard deviation. Statistical significance was accepted at P < 0.05.

	Group 1 (N4RF_NO) N = 20	Group 2 (N4RF_O) N = 35	Group 3 (4RF_NO) N = 9	Group 4 (4RF_O) N = 19		P value
**Gender**	17 Females 3 Males	19 Females 6 Males	5 Females 4 Males	21 Females 7 Males		0.402
**Age (Years)**	62 ± 6	58 ± 11	65 ± 5	65 ± 11		0.135
	**Group 1**	**Group 2**	**Group 3**	**Group 4**	**Group Comparisons**	
**Diastolic blood pressure** **(mmHg)**	71.5 ± 7	71.5 ± 6	82.4 ± 9	83.9 ± 12.5		
**Systolic blood pressure** **(mmHg)**	123 ± 7.7	122 ± 6.3	170.2 ± 11.2	159 ± 17.2		
	
	
					1–3	<0.05
					1–4	<0.05
**Fasting glucose** **(mmol/L)**	4.9 ± 0.4	5.1 ± 0.3	6.3 ± 0.8	6.8 ± 1.3	2–3	<0.05
**Blood triglycerides** **(mmol/L)**	0.9 ± 0.3	1.1 ± 0.3	2.1 ± 0.4	2.5 ± 1.0	2–4	<0.05
	
	
	
**Blood high density lipoprotein cholesterol** **(mmol/L)**	1.8 ± 0.4	1.7 ± 0.3	1.0 ± 0.2	1.0 ± 0.2		
**Waist circumference** **(cm)**	72.9 ± 7	86.6 ± 5	80.6 ± 4.5	92.6 ± 10.2	1–2	<0.05
					1–4	<0.05
					2–3	<0.05
					2–4	<0.05

### Circulatory levels of TNF-α and leptin exacerbated, and adiponectin decreased in people with central obesity and the clustering of other 4 MetS risk factors

**Fig 2:**
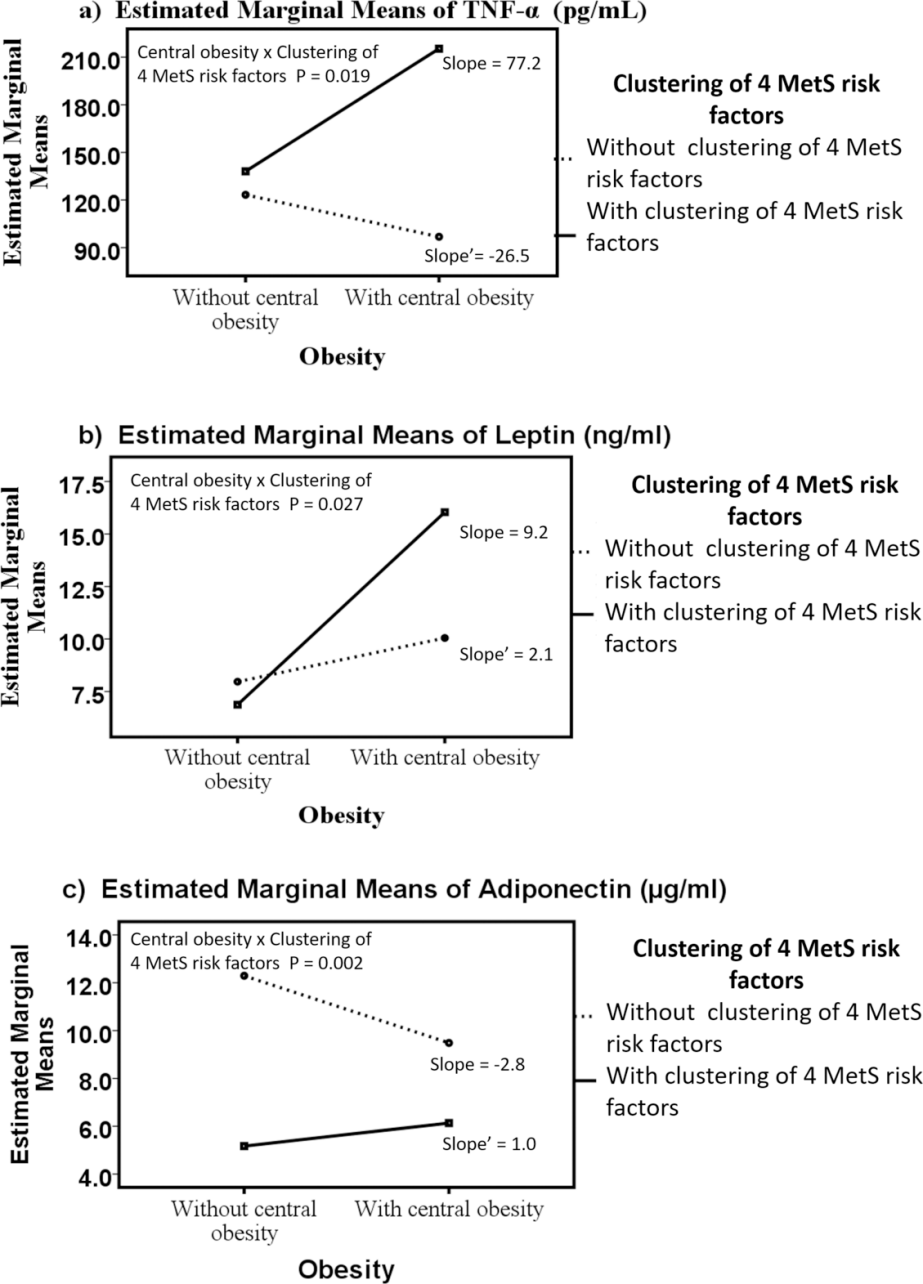
The interaction of central obesity with the clustering of the other 4 MetS risk factors on adipokines. The line graphs represent the direction and slope of interaction effect of central obesity and the clustering of the other 4 MetS risk factors (high fasting blood glucose, high triglycerides, low HDL and high systolic and diastolic BP) on adipokines including TNF-α (A), leptin (B) and adiponectin (C) in Hong Kong Chinese women categorized into four groups: 1) subjects with none of the cardiometabolic risk factors (N4RF_NO; n = 20), 2) subjects with only central obesity without the other 4 MetS cardiometabolic risk factors (N4RF_O; n = 35), 3) subjects without central obesity but with the other 4 MetS cardiometabolic risk factors (4RF_NO; n = 9), and 4) subjects with all five MetS cardiometabolic risk factors (4RF_O; n = 19). Data are expressed in estimated marginal means. Statistical significance was accepted at P < 0.05.

Interaction effects between central obesity and the clustering of the other 4 MetS cardiometabolic risk factors were found for TNF-α (Wald chi square = 5.47, P = 0.019; Fig 2A), leptin (Wald chi square = 4.92, P = 0.027; Fig 2B) and adiponectin (Wald chi square = 9.17, P = 0.002; Fig 2C). After adjusting for age and sex as covariates, the interaction effect was still significant for TNF-α, leptin and adiponectin.

Furthermore, the four-group comparisons indicated that the 4RF_O group had a 74% higher TNF-α level (mean difference = 92 pg/ml, P = 0.048), 101.3% higher leptin level (mean difference = 8 ng/ml, P < 0.001), and 50% lower adiponectin level (mean difference = - 6.2 μg/ml, P < 0.001) than the N4RF_NO group (i.e., difference between central obese subjects with 4RF and non-central obese subjects without 4RF). The 4RF_O group showed a 122% higher TNF-α level (mean difference = 118.4 pg/ml, P < 0.001) and 35.3% lower adiponectin level (mean difference = - 3.4 μg/ml, P = 0.006) compared with the N4RF_O group (i.e., difference between the presence and the absence of the clustering of 4 cardiometabolic risk factors in central obese subjects). The 4RF_O had a 133% higher leptin level (mean difference = 9.2 ng/ml, P = 0.023) compared with the 4RF_NO group (i.e., the difference between central obesity and non-central obesity in the 4RF subjects). The 4RF_NO group had a 57% lower adiponectin level (mean difference = - 7.1 μg/ml, P < 0.001) than the N4RF_NO group (i.e., the difference between the presence and the absence of the clustering of 4 cardiometabolic risk factors in non-central obese subjects) (Table 2).

**Table 2:** Table represents the serum concentrations of proinflammatory and anti-inflammatory adipokines and insulin hormone in the following 4 groups: 1) subjects with none of the cardiometabolic risk factors (N4RF_NO; n = 20), 2) subjects with only central obesity without the other 4 MetS cardiometabolic risk factors (N4RF_O; n = 35), 3) subjects without central obesity but with the other 4 MetS cardiometabolic risk factors (4RF_NO; n = 9), and 4) subjects with all five MetS cardiometabolic risk factors (4RF_O; n = 19). The five MetS cardiometabolic risk factors include central obesity, high fasting blood glucose, high triglycerides, low HDL cholesterol and high systolic and diastolic BP. Data are expressed as mean ± standard deviation, 95% confidence intervals (CI) [X1 (lower bound), X3 (upper bound)]. Statistical significance was accepted at P < 0.05.

		Concentration (mean ± SD), 95% CI [X1, X3]				Group comparisons	Crude P value	Adjusted P value
		Group 1	Group 2	Group 3	Group 4			
Group name (sample size)		N4RF_NO (n = 20)	N4RF_O (n = 35)	4RF_NO (n = 9)	4RF_O (n = 19)			
**Proinflammatory** **adipokines**	**TNF-α** (pg/ml)	123.3 ± 81.6, [88.4, 158.2]	96.8 ± 162.0, [35.8, 157.9]	138.1 ± 34.6, [116.8, 159.4]	215.3 ± 127.8, [168.8, 261.8]	1–4	0.008	0.048
						2–4	<0.001	<0.001
	**Leptin** (ng/ml)	7.9 ± 9.2, [4.0, 11.9]	10.0 ± 5.2, [8.0, 12.0]	6.8 ± 3.9, [4.9, 9.3]	16.0 ± 10.3, [12.3, 19.8]	1–4	<0.001	<0.001
						3–4	0.004	0.023
	**Chemerin** (ng/ml)	69.9 ± 11.9, [64.7, 74.9]	82.0 ± 17.5, [75.5, 88.7]	101.2 ± 23.7, [86.6, 115.8]	112.3 ± 35.6, [99.4, 125.3]	1–4	<0.001	<0.001
						1–3	0.001	0.002
						2–4	<0.001	0.001
	**IL-8** (pg/ml)	69.2 ± 81.7, [34.3, 104.1]	79.2 ± 236.1, [-9.8, 168.2]	212.7 ± 74.5, [166.9, 258.6]	118.3 ± 88.3, [86.2, 150.4]	1–3	<0.001	0.001
						2–4	<0.001	0.001
						2–3	<0.001	<0.001
	**IL-6** (pg/ml)	6.5 ± 4.6, [4.5, 8.4]	17.1 ± 26.2, [7.2, 26.9]	10.0 ± 4.0, [7.5, 12.5]	14.9 ± 9.7, [11.3, 18.4]	1–4	<0.001	<0.001
						2–4	0.002	0.009
	**PAI-1** (pg/ml)	1201.0 ± 1427.5, [591.2, 1810.7]	1613.1 ± 821.3, [1303.5, 1922.6]	1213 ± 896.7, [661.0, 1765.6]	2010.7 ± 1192.5, [1576.9, 2444.5]	1–4	<0.001	0.001
	**Visfatin** (pg/ml)	772.3 ± 623.8, [505.8, 1038.7]	1129.8 ± 803.7, [826.8, 1432.7]	1384.4 ± 1241.8, [619.5, 2149.3]	1873.0 ± 1163.3, [1449.9, 2296.2]	1–4	<0.001	<0.001
						2–4	0.004	0.025
	**Resistin** (ng/ml)	8.0 ± 2.4, [6.9, 9.0]	8.6 ± 3.2, [7.4, 9.8]	8.4 ± 2.8, [6.6, 10.2]	10.7 ± 5.4, [8.8, 12.7]	No significant difference		
	**CCL2** (pg/ml)	225.6 ± 60.9, [199.6, 251.6]	224.5 ± 90.5, [190.4, 258.7]	243.6 ± 48.8, [213.5, 273.6]	226.4 ± 55.5, [206.2, 246.6]	No significant difference		
**Anti-inflammatory** **adipokines**	**Adiponectin** (μg/ml)	12.3 ± 3.5, [10.8, 13.7]	9.5 ± 4.1, [7.9, 11.0]	5.2 ± 1.1, [4.5, 5.9]	6.1 ± 2.5, [5.2, 7.0]	1–3	<0.001	<0.00
						1–4	<0.001	<0.001
						2–4	0.001	0.006
						3–2	0.001	0.005
	**IL-10** (pg/ml)	39.0 ± 20.9, [30.1, 47.9]	32.3 ± 12.9, [27.4, 37.2]	53.8 ± 70.2, [10.5, 97.0]	67.4 ± 69.1, [42.3, 92.5]	No significant difference		
**Hormone**	**Insulin** (pg/ml)	235.8 ± 77.5, [202.7, 268.9]	305.8 ± 94.1, [270.3, 341.3]	300.3 ± 57.4, [264.9, 335.7]	387.2 ± 117.1, [344.6, 4429.8]	1–4	<0.001	<0.001
						1–2	0.005	0.031

### Circulatory levels of insulin, chemerin, IL-6 and PAI-1 exacerbated in people with only central obesity

**Fig 3:**
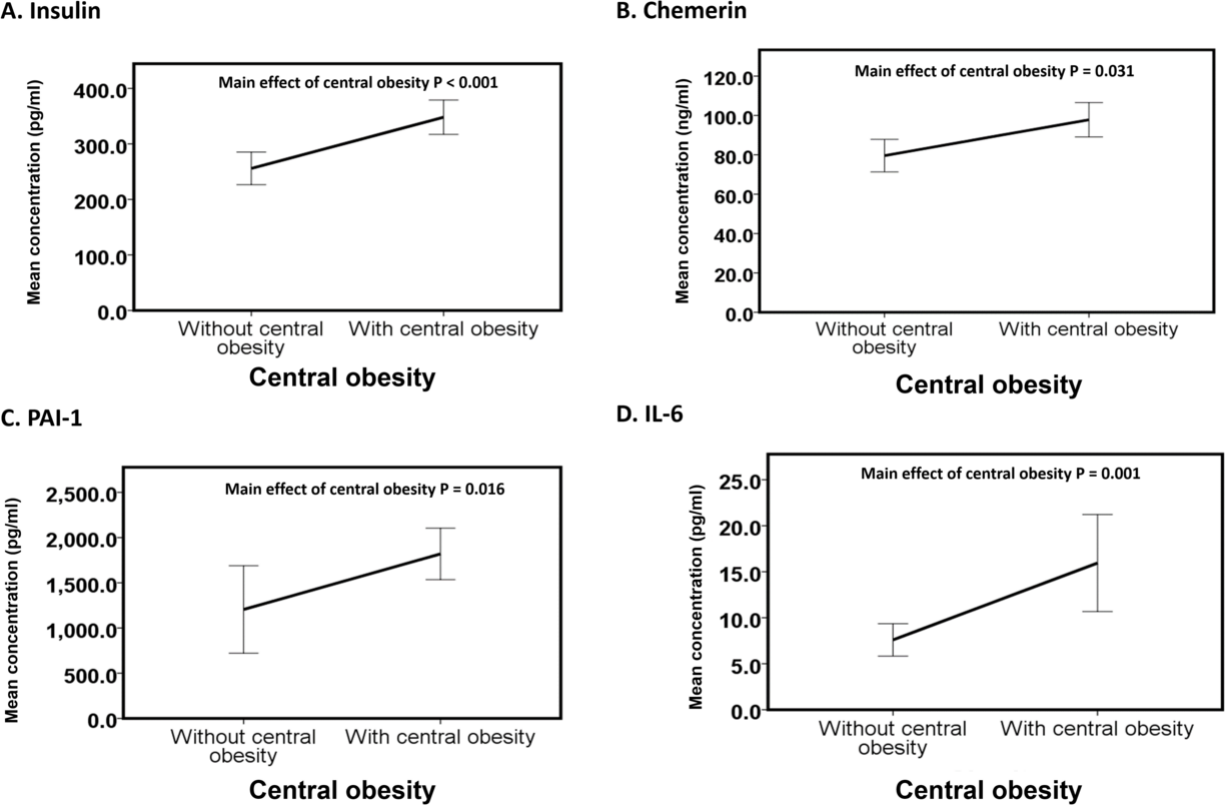
Main effect of obesity on adipokines. The line graphs (A-D) represent the means of insulin, chemerin, IL-6, and PAI-1 of subjects without central obesity (n = 29) versus subjects with central obesity (n = 54) irrespective of the presence of the clustering of 4 MetS risk factors (high fasting blood glucose, high triglycerides, low HDL and high systolic and diastolic BP). The data are expressed as the mean ± 1 standard deviation. Statistical significance was accepted at P < 0.05.

The main effects of central obesity were observed on insulin (Wald chi square = 17.4, P < 0.001, Fig 3A), chemerin (Wald chi square = 4.7, P = 0.031, Fig 3B), IL-6 (Wald chi square = 7.6, P = 0.001, Fig 3C), and PAI-1 (Wald chi square = 5.9, P = 0.016, Fig 3D). We found increases (mean difference ± standard error) in insulin (78.5 ± 18.8 pg/ml), chemerin (117.2 ± 54.2 ng/ml), PAI-1 (604.7 ± 250.1 pg/ml) and IL-6 (7.7 ± 2.8 pg/ml) levels in obese subjects compared to non-obese subjects (Fig 3A, 3B, 3C and 3D).

### Circulatory levels of insulin, chemerin, IL-8 and Visfatin exacerbated in individuals with only the clustering of other 4 MetS risk factors

**Fig 4:**
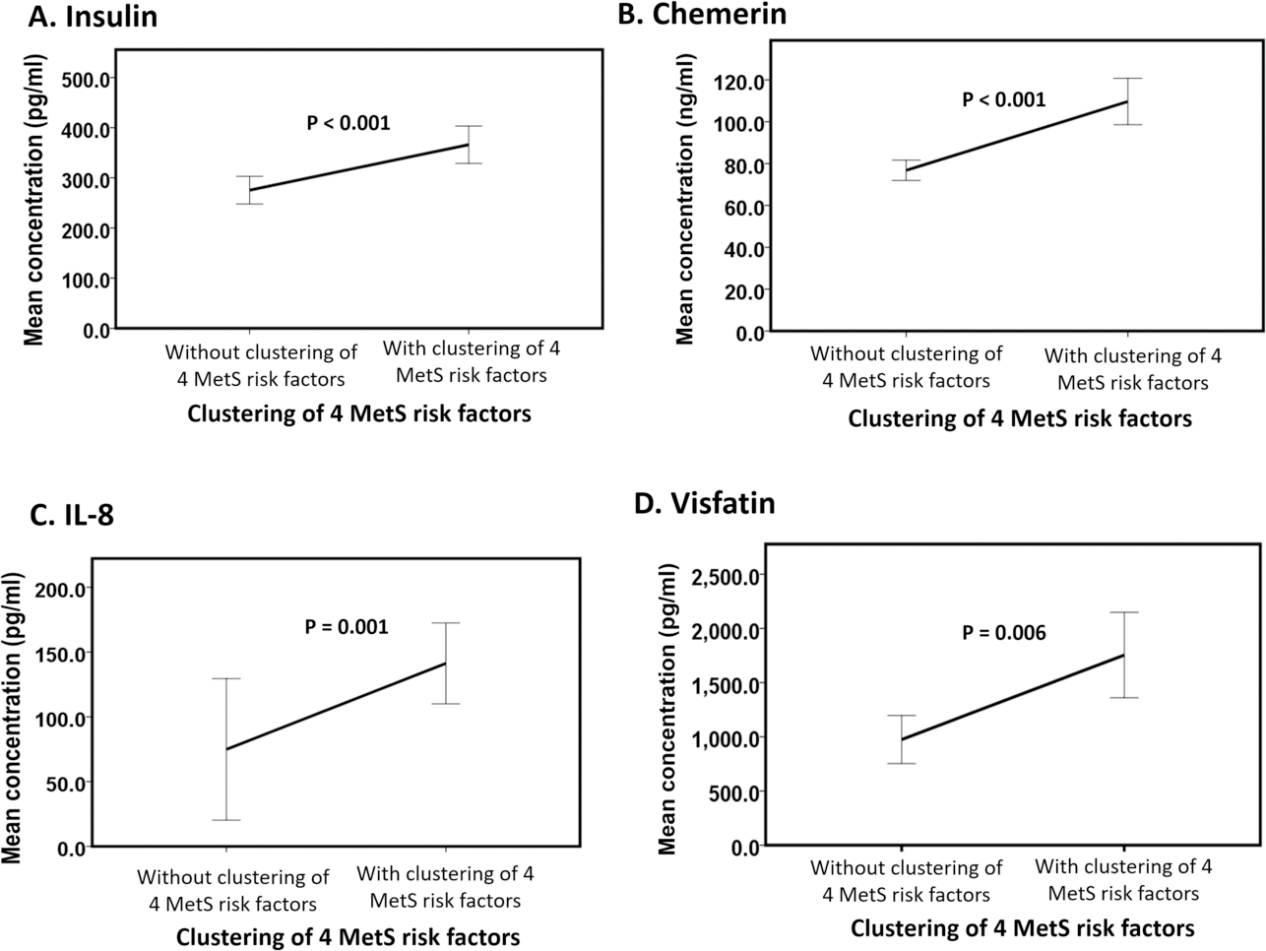
Main effect of the clustering of 4 MetS risk factors on adipokines. The line graphs (A-D) represent the means of insulin, chemerin, IL-8, and visfatin of subjects without the clustering of 4 MetS risk factors (high fasting blood glucose, high triglycerides, low HDL and high systolic and diastolic BP) (n = 46) versus subjects with the clustering of 4 MetS risk factors (n = 37) irrespective of the presence or absence of central obesity. The data are expressed as the mean ± 1 standard deviation. Statistical significance was accepted at P < 0.05.

The main effects of the clustering of 4 MetS cardiometabolic risk factors were observed on insulin (Wald chi square = 15.1, P < 0.001, Fig 4A), chemerin (Wald chi square = 32.3, P < 0.001 Fig 4B), IL-8 (Wald chi square = 10.4, P = 0.001, Fig 4C), and visfatin (Wald chi square = 7.6, P = 0.006, Fig 4D). We found increases (mean difference ± standard error) in insulin (73 ± 18.8 pg/ml), chemerin (30.8 ± 54.2 ng/ml), IL-8 (91.3 ± 28.3 pg/ml) and visfatin (677.7 ± 245.6 pg/ml) levels in subjects with the clustering of all 4 MetS cardiometabolic risk factors compared to subjects without the clustering of 4 MetS cardiometabolic risk factors (Fig 4A, 4B, 4C & 4D).

### Correlation between MetS risks factors and adipokines

Chemerin (R = 0.62), insulin (R = 0.53), visfatin (R = 0.39), PAI-1(R = 0.34), leptin (R = 0.27) and resistin (R = 0.14) were positively correlated whereas adiponectin (R = - 0.69) and IL-10 (R = - 0.024) were negatively correlated with fasting glucose level. Insulin (R = 0.53), chemerin (R = 0.49), PAI-1(R = 0.44), leptin (R = 0.33), visfatin (R = 0.30), TNF-α (R = 0.18), and IL-10 (R = 0.13) were positively correlated whereas adiponectin (R = - 0.48) was negatively correlated with waist circumference. Chemerin (R = - 0.59), TNF-α (R = - 0.44), insulin (R = - 0.42), visfatin (R = - 0.31), PAI-1 (R = - 0.24), and resistin (R = - 0.23) were negatively correlated whereas adiponectin (R = 0.74) was positively correlated with HDL-cholesterol. Chemerin (R = 0.58), insulin (R = 0.44), visfatin (R = 0.41) and IL-10 (R = 0.038) were positively correlated whereas adiponectin (R = - 0.62) was negatively correlated with triglycerides. Chemerin (R = 0.30) and insulin (R = 0.29) were positively correlated whereas adiponectin (R = - 0.48) was negatively correlated with systolic BP. Insulin (R = 0.27) was positively correlated whereas adiponectin (R = -0.28) was negatively correlated with diastolic BP (S1 Fig).

## Discussion

Metabolic and cardiovascular complications are major obesity-associated burdens, and thus recognising obese people at threat for MetS is a foremost healthcare priority. The enduring worldwide obesity epidemic is a significant threat to the population and healthcare systems owing to the related morbidity and high treatment costs [38]. The prevalence of obesity has been increased manifold in most of the Asian countries in the past few decades [39–42]. Western Pacific region and South East Asia are faced with widespread diseases associated with obesity such as diabetes and CVD [42]. Some countries like Indonesia and Vietnam are in the early stages of development of diseases associated with obesity while others like Hong Kong, Singapore, Malaysia, and Japan are at more advanced stages [8]. Therefore, the present study was designed to examine the interaction of central obesity with the clustering of the other 4 MetS risk factors (including high BP, high triglycerides, high fasting blood glucose and reduced HDL cholesterol) on circulatory proinflammatory and anti-inflammatory adipokines in Hong Kong Chinese adults. Our results indicated that the interaction effects between central obesity and the clustering of other 4 MetS cardiometabolic risk factors existed for TNF-α, adiponectin and leptin. TNF-α increased, and adiponectin decreased in centrally obese subjects that exhibited a clustering of 4 risk factors compared to central obese subjects without this clustering. Leptin was increased in centrally obese subjects, with the clustering of 4 MetS risk factors compared to subjects without the clustering of 4 MetS risk factors. For subjects with the clustering of 4 risk factors, leptin was increased in centrally obese subjects when compared to non-centrally obese subjects.

### Exacerbated circulatory proinflammatory (TNF-α and leptin), and decreased anti-inflammatory (adiponectin) adipokine levels in individuals with central obesity and the clustering of other 4 MetS risk factors might be associated with the risk of T2D

TNF-α is a cell signalling protein that is responsible for metabolic imbalances such as insulin resistance and altered lipid and carbohydrate metabolism [43,44]. For subjects with central obesity, the clustering of 4 risk factors was associated with an increase in TNF-α concentration. Although the exact molecular mechanisms that link obesity to other MetS risk factors remains unclear, TNF-α has been shown to be associated with MetS risk factors [45,46]. Furthermore, TNF-α has been reported to be increased in proportion to the number of the MetS components [45,47]. Increases in circulating TNF-α has also been suggested to be related to insulin resistance and augmented systemic inflammation [48,49]. Apart from insulin resistance, obesity-associated hypertension led to an increase in TNF-α in a French Canadian cohort [47]. Similarly, subjects with severe hyperinsulinemia, hypertriglyceridemia, high body mass index, and low HDL-cholesterol were reported to have high circulating TNF-α levels [45,50]. Increases in circulating TNF-α are associated with peripheral insulin resistance, increased plasma glucose and insulin levels before the onset of T2D, whereas the increased fasting glucose level induced by obesity can be prevented by TNF-α deficiency [45].

In addition, our data revealed the interaction effect between central obesity and the clustering of the other 4 MetS risk factors on adiponectin and leptin. We observed relatively lower circulating adiponectin in centrally obese subjects with the clustering of 4 MetS risk factors as compared to the subjects with only central obesity, with the clustering of 4 MetS risk factors or without any MetS risk factors. Consistent with our results, a low circulating adiponectin level has been previously shown to correlate with an increase in MetS risk factors [51]. Similarly, hypoadiponectinemia has been suggested to place MetS patients at a higher risk of developing T2D [52]. Adiponectin activates 5' adenosine monophosphate-activated protein kinase (AMPK) [53], which regulates lipogenesis and cholesterol synthesis [54]. Cross-sectional studies have demonstrated that hypoadiponectinemia might be an independent risk factor for hypertension [55,56]. The renin-angiotensin system (RAS) plays an important role in the regulation of BP and cardiovascular function. This system is activated in MetS and leads to arterial wall inflammation, elevation of the angiotensin II level, and oxidative stress [57,58]. Suppressing RAS by elevating adiponectin has been proposed as an effective strategy for treating MetS [59]. In addition to adiponectin, we also observed relatively higher leptin in centrally obese subjects with the clustering of 4 MetS risk factors compared to the subjects with only central obesity, with the clustering of 4 MetS risk factors or without any MetS risk factors. Consistently, high circulating leptin level has been observed in individuals with MetS risk factors compared to those without MetS risk factors [60]. The exacerbation in leptin level in obese patients with the clustering of 4 risk factors indicates that leptin may be involved in the severity of MetS and may lead to T2D [61,62] associated with central obesity. The inclined pattern of serum leptin has been reported in MetS. Specifically, associations between leptin and other risk factors of MetS (r ≥ ± 0.1) and body mass index (r ≥ ± 0.5) for both sexes have been established [63]. In contrast, serum leptin has been reported to be increased in proportion to the number of MetS risk factors, regardless of the weight status of the subjects [64]. The contradictory findings between studies may be explainable by the complexity of the definition of MetS; for instance, an identical number of MetS risk factors might not indicate that the subjects would share precisely the same cardiometabolic risk factor profiles and characteristics.

The interaction between the components of the clinical phenotype (e.g., central obesity, insulin resistance, hypertension, and dyslipidemia) of MetS might contribute to the development of a pro-inflammatory state [65]. Previous studies have suggested that the anti-inflammatory: pro-inflammatory adipokine equilibrium is important for preventing MetS, especially in central obese subjects [34]. For instance, a low adiponectin: leptin ratio indicates the severity of MetS in patients with central obesity compared to those without MetS [35]. Additionally, the adiponectin: leptin ratio has been shown to correlate with variations in systolic BP, insulin sensitivity, total cholesterol and low-density lipoprotein in MetS patients with obesity [35]. Consistently, our results also indicate the interaction effect between central obesity and the clustering of 4 MetS risk factors on the adiponectin: leptin ratio (P = 0.016) (data not shown). Physiologically, a disproportion in the expression of anti-/pro-inflammatory adipokines results in an increased size of adipocytes. These hypertrophied adipocytes result in a shift towards the dominance of pro-inflammatory adipokines [66]. The imbalance in the production of pro-inflammatory and anti-inflammatory biomolecules precedes increased immune cell infiltration and the induction of a macrophage phenotype switch in visceral adipose tissue [67]. We propose that the interaction between central obesity and the clustering of all 4 MetS risk factors would favour a dominance of pro-inflammatory adipokines that might be involved in increasing the severity of MetS.

### Elevated circulating levels of insulin, chemerin, PAI-1 and IL-6 might represent the first-line adipokines that initiate subsequent inflammatory cascades in individuals with only central obesity

The main effect of central obesity was observed in insulin, PAI-1, chemerin and IL-6. Interestingly, all 4 biomarkers were shown to be augmented during the differentiation of preadipocytes to adipocytes. Hyperinsulinemia enhances the differentiation of preadipocytes to adipocytes, which is a major contributor to the increased PAI-1 in obese subjects [68]. Chemerin is a chemoattractant recruiting macrophages to adipose tissue. Furthermore, adipocytes serve as both secretary cells of chemerin and a target for autocrine chemerin signaling, regulation of adipogenesis and adipocyte differentiation [69]. Similarly, several studies have reported that serum IL-6 is elevated in obese patients [70] and that IL-6 is highly expressed in preadipocytes compared with the mature adipocytes of obese mice [71]. Our results show that increases in adipokines (PAI-1, IL-6, and chemerin), which favour the differentiation of preadipocytes attributed to the effect of central obesity, might represent the first-line adipokines that initiate subsequent inflammatory cascades in individuals with only central obesity.

### Elevated circulating levels of insulin, chemerin, IL-8 and visfatin in individuals with only the clustering of 4 MetS risk factors might result from the migration and activation of macrophages

On the other hand, insulin, chemerin, IL-8 and visfatin demonstrated the main effect of the clustering of 4 MetS risk factors. Insulin resistance has been reported as a combination of macrophages accumulation that secretes proinflammatory adipocytokines and altered outcome of insulin target cells [72]. Notably, in this context, the primary sources of the above adipokines are macrophages, implying that migration and activation of macrophages may be the key events that mediate the development of MetS. The migrated macrophages secrete chemotactic cytokines such as IL-8 that further induce macrophage migration to adipose tissue. Visfatin is produced in visceral adipose tissue and has been proposed as the missing link between intra-central obesity and diabetes [73]. Visfatin is secreted by neutrophils and is regulated by pro-inflammatory factors (such as TNF-α and IL-6) in monocytes [74,75]. Likewise, chemerin is a chemokine that recruits macrophages to adipose tissue and plays an important role in pro-inflammatory processes such as chemotaxis modulation and dendritic cell and macrophage activation [76]. Researchers have verified that the serum levels of chemerin are higher in MetS patients compared with controls and are correlated with the level of the MetS components [77]. The adipokines mentioned above have been reported to increase the severity of MetS; however, the adipokine expression levels in MetS patients with all 4 MetS risk factors have not been reported. Therefore, our results suggest that changes in insulin, IL-8, visfatin and chemerin levels may indicate the severity of MetS with the clustering of 4 MetS risk factors. These adipokines recruit pro-inflammatory macrophages and, hence, promote the transition from a mild metabolic dysfunction phenotype to a full metabolic dysfunction phenotype [78].

### Individuals with different metabolic characteristics possessed distinct adipokine profiles: A group wise comparison

We compared the levels of adipokines among lean subjects with none of the MetS cardiometabolic risk factors (N4RF_NO), subjects without the clustering of 4 MetS cardiometabolic risk factors but with central obesity (N4RF_O), subjects with the clustering of 4 MetS cardiometabolic risk factors but without central obesity (4RF_NO), and subjects with the clustering of 4 MetS cardiometabolic risk factors and central obesity (4RF_O). In line with our hypothesis, subjects with different metabolic characteristics possessed distinct adipokine profiles.

Central obesity is very often the first risk factor that patients acquire during the development of MetS [79]. After acquiring the central obesity, other risk factors begin to emerge. Therefore, adipokine profiles in three distinct groups (N4RF_NO, N4RF_O and 4RF_O) in the present study can give us some hints about the classical progression of MetS. It came as no surprise that subjects in N4RF_NO had the lowest level of all adipokines, except adiponectin. Our results clearly demonstrated that insulin, leptin, chemerin and PAI-1 were elevated and adiponectin was reduced in N4RF_O subjects when compared to N4RF_NO subjects (Table 2). As a result of increased adipose depot, adipose tissues in N4RF_O subjects were possibly infiltrated by a large number of macrophages which further stimulated the secretion of adipokines by both adipocytes and activated macrophages, resulting in a substantial increase in TNFα, IL-8, insulin, chemerin and visfatin and a decrease in adiponectin in 4RF_O subjects compared to N4RF_O. It is worth to note that six out of twelve biomarkers were significantly altered in subjects from N4RF_O compared to 4RF_O. Among the six biomarkers, four of them, including insulin, adiponectin, TNFα, and chemerin, showed the stepwise increase or decrease (for adiponectin) (i.e., from N4RF_NO compared to N4RF_O and then from N4RF_O compared to 4RF_O). These four biomarkers together with macrophages and adipocytes can create a positive feedback loop that further amplifies inflammation in adipose tissue and induces systemic inflammation [79]. It is possible that obesity induces altered metabolism and gene expression in enlarged adipocytes, leading to increased lipolysis and release of chemerin. As a result, a large number of macrophages migrate into adipose tissue due to chemotactic effect. Hyperinsulinemia promotes TNFα production in migrated macrophages [72]. Finally, TNFα downregulates the anti-inflammatory adiponectin [80] and modulates macrophage polarization to an unfavorable M1 phenotype [81], resulting in a pro-inflammatory microenvironment in adipose tissue and further stress on adipocytes. Therefore, we speculate that these four biomarkers are the key players in promoting adipose tissue inflammation, necrosis, and systemic inflammation. Our correlation analysis further confirmed the importance of these four biomarkers. Both insulin and adiponectin were correlated with all manifestations of MetS. Chemerin and TNFα were associated with five and two signs, respectively, of MetS. Most importantly, systolic blood pressure, waist circumference, triglycerides, HDL and glucose had the strongest correlations with adiponectin and chemerin. Our results indicated that, among the 12 studied biomarkers, adiponectin, chemerin, insulin and TNFα showed a progressive increase/decrease (i.e., N4RF_NO compared to N4RF_O compared to 4RF_O). Three biomarkers including adiponectin, chemerin and insulin correlated with at least 5 out of 6 manifestations of MetS. Surprisingly, both adiponectin and chemerin had the strongest association with all criteria of MetS, except for diastolic blood pressure. This study provides comprehensive profiling data in investigating the association between adipokines and manifestations of MetS. Our results suggested that adiponectin and chemerin might be the most important biomarkers in the development of MetS related to obesity.

Although MetS is strongly associated with adiposity, subjects with MetS are unnecessarily obese. The term metabolically obese but normal weight has been used to describe this particular group of individuals who possess normal body weight but display insulin resistance, increased abdominal adiposity, higher levels of blood pressure and abnormal lipid profiles [82]. In the current study, subjects from 4RF_NO manifested an unfavorable adipokine profile. Chemerin, IL-8, and insulin were significantly increased whereas adiponectin was significantly decreased when compared to subjects from N4RF_NO. Consistent with our previous observation, chemerin and adiponectin are strongly associated with MetS regardless of the body weight. Interestingly, adipokines that were higher in subjects from 4RF_O, including TNFα, visfatin, and IL-6, were not found elevated in subjects from 4RF_NO. Our results implied that different mechanisms possibly mediate the developments of MetS in obese and non-obese subjects. Importantly, IL-8 was significantly higher in subjects from 4RF_NO. IL-8 is a macrophage-derived mediator of angiogenesis [83] and involved in the development of MetS, but its exact role in obesity and development of MetS remains to be investigated.

## Conclusion

In this study, our findings are based on the screening of insulin and 11 adipokines to examine the interaction of central obesity and the clustering of the other 4 MetS risk factors on circulatory proinflammatory and anti-inflammatory adipokines. Therefore, a study profiling all adipokines for these clinical and pathophysiological changes to improve early patient identification may be warranted. Although the adopted sample size has resulted in a statistical power of 80% or above for most of our significant outcome measures, the constraint of sample size might also be a limitation in the present study. Furthermore, the present investigation was limited to an Asian population; hence our results cannot be generalized to non-Asian populations. In conclusion, our results revealed the interaction of central obesity and the clustering of the other MetS cardiometabolic risk factors for exacerbating circulatory TNF-α and leptin; and reducing adiponectin. This combined effect of central obesity with all of the 4 MetS risk factors increases the proinflammatory status of adipokines. These altered adipokine patterns (low circulating adiponectin and high TNF-α and leptin) may provide useful information on the additional risk of metabolic derangements. Our results indicate that the inflammatory status of MetS disorders might become severe in the presence of central obesity. These results also confirm the International Diabetes Federation’s definition of MetS by placing central obesity as the compulsory risk factor for MetS diagnosis. Further research is needed to fully understand the exact roles of adipokines in the progression of MetS and the subsequent development of chronic diseases such as T2D and CVD.

## Supporting information

S1 Fig Group wise correlation scatterplot of all twelve biomarkers with all six characteristics of MetS. Statistical significance was accepted at P ≤ 0.05 and indicated by “* “and “R” represents the spearman correlation coefficient.
(TIF)
