# Supplementary figures and images for: Adipokines demonstrate the interacting influence of central obesity with other cardiometabolic risk factors of metabolic syndrome in Hong Kong Chinese adults
Source: PLoS One. 2018 Aug 16;13(8):e0201585. doi: 10.1371/journal.pone.0201585 (PMC6095502; doi:10.1371/journal.pone.0201585)

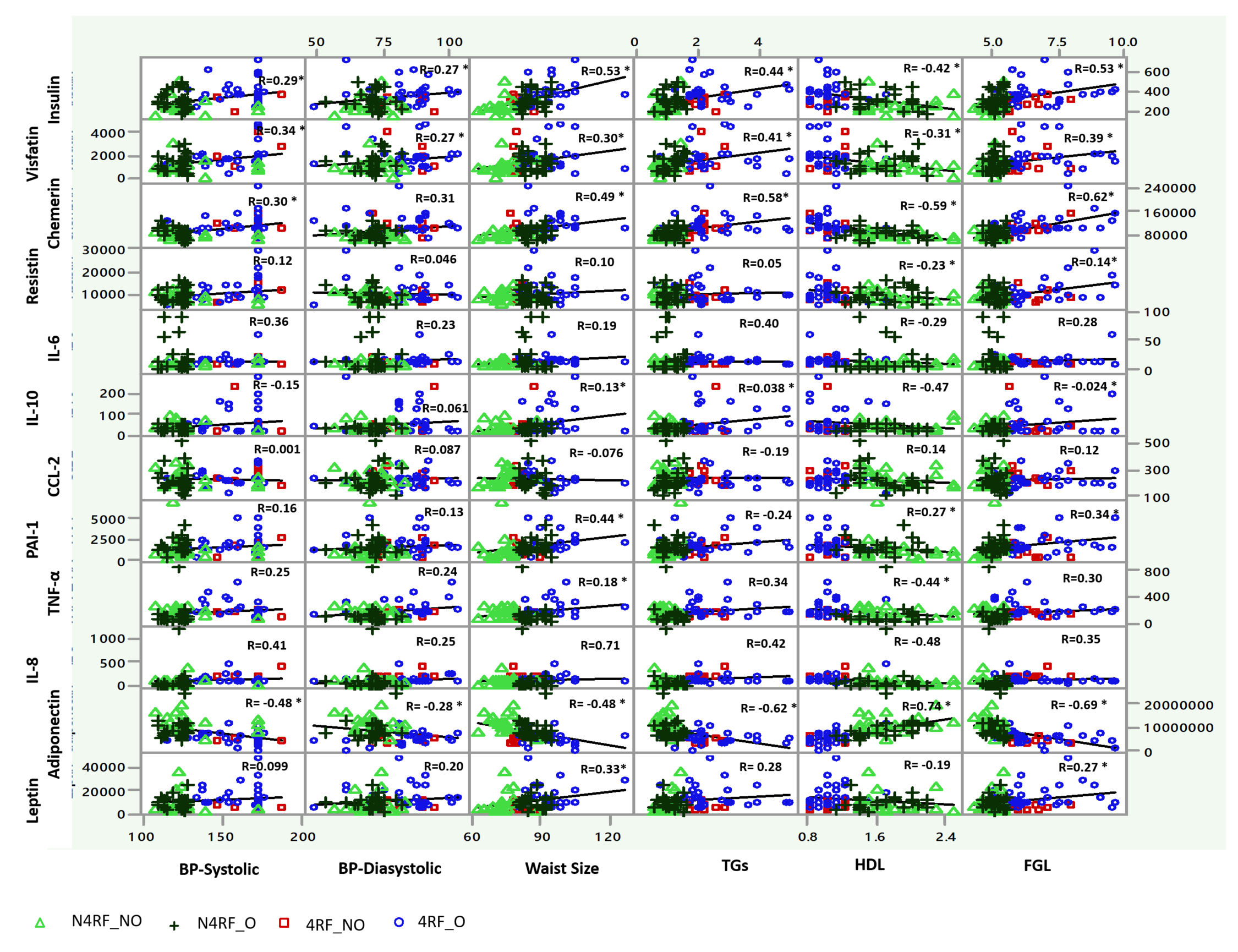

Supplement: S1 Fig — Statistical significance was accepted at P ≤ 0.05 and indicated by “* “and “R” represents the spearman correlation coefficient. (TIF) [file pone.0201585.s001.tif]
